# Clinical and genetic characteristics of pulmonary arterial hypertension in Lebanon

**DOI:** 10.1186/s12881-018-0608-7

**Published:** 2018-05-30

**Authors:** Ossama K. Abou Hassan, Wiam Haidar, Georges Nemer, Hadi Skouri, Fadi Haddad, Imad BouAkl

**Affiliations:** 1 Department of Internal Medicine, Faculty of Medicine, American University of Beirut, P.O.Box: 11-0236, Beirut, Lebanon; 2 Department of Biochemistry and Molecular Genetics, Faculty of Medicine, American University of Beirut, P.O.Box: 11-0236, Beirut, Lebanon; 3 Univeriste St Jospeh, Beirut, Lebanon

**Keywords:** Pulmonary hypertension, *BMPR2*, Mutation

## Abstract

**Background:**

Pulmonary arterial hypertension (PAH) is a rare disease with an incidence rate of 2–6 cases per million per year. Our knowledge of the disease in the Middle East and North Africa (MENA) region is limited by the small number of clinical studies and the complete absence of genetic studies.

**Methods:**

Our aim was to shed light on the clinical and genetic characteristics of PAH in Lebanon and the region by using exome sequencing on PAH patients referred to the American University of Beirut Medical Center (AUBMC). Twenty-one idiopathic, hereditary and Congenital Heart Disease (CHD) PAH patients were prospectively recruited, their clinical data summarized, and sequencing performed.

**Results:**

The mean age at diagnosis was 33 years with a female preponderance of 70%. The mean pulmonary artery pressure at the time of diagnosis was 55. Genetic testing showed that 5 out of 19 idiopathic and Congenital Heart Disease PAH patients had *Bone Morphogenetic Protein Receptor 2* (*BMPR2)* mutations at 25% prevalence, with 2 of these patients exhibiting a novel mutation. It also showed the presence of 1 *BMPR2* mutation with 100% penetrance in a heritable PAH family. In the remaining cases, the lack of a complete genotype/phenotype correlation entailed a multigenic inheritance; suspected interactions involved previously associated genes *T-box transcription factor 4 (TBX4), Bone Morphogenic Protein 10 (BMP10)* and *Growth Differentiation Factor 2 (GDF2)*.

**Conclusions:**

This is the first study that looks into the genetic causes of PAH, including known and new *BMPR2* mutations, in the MENA region. It is also the first study to characterize the clinical features of the disease in Lebanon.

**Electronic supplementary material:**

The online version of this article (10.1186/s12881-018-0608-7) contains supplementary material, which is available to authorized users.

## Background

Pulmonary Arterial Hypertension (OMIM #178600) is a devastating disorder of the small pulmonary arteries. It is characterized by progressive neo-intimal proliferation, smooth muscle hypertrophy and occlusive vascular lesions leading to increased vascular resistance and pressure [1]. It is a rare disease with prevalence estimated to be between 15 and 60 cases per million [2]. Males are at less risk than females with a 2–3:1 female: male ratio [2, 3].

Early PAH registries in the eighties provided valuable information on the baseline characteristics and outcomes of the disease and helped with the understanding of the natural progression of PAH [4]. Patients in early registries were younger with a mean age of 36 and with female preponderance. Data from more recent European and North American registries suggest that PAH patients are becoming older and have a better survival [5, 6]. A longitudinal observational study on PAH incidence in the United Kingdom and Ireland spaning a 9-year-period noticed an increase in the age of diagnosis of PAH patients from 45 to 52 years over the same period. These changes in demographics may reflect not only an improvement in survival but also better referral practices in these countries [7]. The incidence, prevalence and burden of disease in the MENA region have never been reported before. Clinical and demographic characteristics of PAH patients have only been documented in a single center registry in Saudi Arabia, and in an online Iranian registry. Both sources show a mean age of 36 years and a 2:1 female preponderance [8, 9].

Despite our growing knowledge of the disease and its progression, PAH patients have significantly reduced survival [10]. Most of drugs used (endothelin receptor antagonists, guanylate cyclase stimulators, prostacyclin analogues and prostacyclin receptor agonists) are prohibitively expensive and not readily available in middle to low-income countries such as Lebanon. This may affect disease severity and prevalence in these countries, making the care of PAH patients even more challenging.

In the year 2000, the *BMPR2* gene was first reported to be linked to the progression of the disease [11]. Around 80% of *hereditary* PAH patients and 25% of *idiopathic* disease patients have *BMPR2* mutations [12, 13]. Twenty-one other genes have also been implicated in the development PAH, with the most frequent mutations occurring in *ALK1*, *ENG*, *SMAD9*, *CAV1*, *KCNK3*, MADH9 and *EIF2AK4* [14]. The penetrance of *BMPR2* mutations ranges between 27 and 50% in females and 14–43% in males [3], depending on the mutation site within the gene [15]. *BMRR2* mutations have also been linked to PAH with congenital heart disease [16]. Trying to understand the low penetrance of *BMPR2* and the yet unidentified genetic causes of the remaining 20% of the hereditary form of PAH, new research strategies have shifted towards using next-generation sequencing (NGS) in the search for additional mutations or compound interactions of known genes [17]. There is a a growing acceptance of a double hit phenomenon – a major gene with an additional modifier gene- supporting the lower penetrance of mutations in genes associated with PAH. As such NGS is poised to be the best technology to unravel this genetic interaction and accounts of all genotype-phenotype discrepancies [18, 19].

This study has many objectives. One objective is to describe the demographic and clinical characteristics of PAH patients in Lebanon. These findings will shed light on the burden of disease and the state of referral and care of patients with PAH in the country. The second objective is to assess for the first time the prevalence and types of genetic mutations in PAH patients in Lebanon and the MENA region. This may help guide future genetic testing, counseling and research in an area that shares common genetic/ethnic background. The final objective is to explore novel genetic mutations or interactions and their implications on the phenotypic manifestations of PAH.

## Methods

### Subject recruitment

Patients diagnosed with PAH at AUBMC between 2015 and 2017 either newly (incidence case) or previously (prevalence case) and patients who were referred to our center were approached for enrollement in the study. They were eligible for enrolment if they had idiopathic PAH, heritable PAH or PAH associated with congenital systemic to pulmonary shunt. All of these conditions are classified under Group 1 PAH, based on the World Health Organization’s (WHO) 2013 guidelines for diagnosis of pulmonary hypertension [2]. Minimal inclusion criteria also included a Right Heart Catheterization (RHC) measuring a mean pulmonary arterial pressure (mPAP) > 25 mmHg at rest and a pulmonary artery wedge pressure (PAWP) < 15mmHG. Following the patient’s consent, clinical data was collected and reviewed, including medical history, physical exam, family history of PAH, and previously performed tests. These tests include pulmonary function test (PFT), chest computerized tomography (CT), CT pulmonary angiography, ventilation perfusion scan and pro-BNP. For those patients not diagnosed and treated at AUBMC, specialist physicians along with our investigators reviewed all medical records for accuracy of diagnosis. Treatment of PAH was also noted. After collection of clinical data, a blood sample was collected from the patients for genetic analysis by whole exome sequencing. Twenty-one PAH patients in total, belonging to 20 families were included in the study. In the case of patients found to have a mutation, non-affected family members were approached for inclusion in the study as controls and blood was collected for sanger analysis of the mutated protein. The study was approved by the institutional review board (IRB) at the American University of Beirut. Genetic analyses and return of genetic data were performed in accordance with protocols approved by the Partners Human Research Committee. Data collection and genetic analysis were performed in accordance with the protocols approved by the IRB at AUBMC.

### Genetic studies

DNA was extracted from peripheral blood. Whole exome libraries were compiled with DNA samples from all affected individuals recruited in this study. The exome was captured using the V5 SureSelect kits from Agilent as per manufacturer’s protocol, and run on the Illumina HiSeq2500. The tests were performed by Macrogen, South Korea. Exome sequences were aligned to hg19 using Novoalign, and variants were called using Genome Analysis Toolkit (GATK). Once variant(s) predicted to be damaging with a minor allele frequency (< 3%) were identified in a patient, Sanger sequencing was performed to confirm thier occurence, and all family members were screened for those particular variant(s). Primers were speficially designed to amplify the region(s) of interest that include those variants, and amplified fragments were analyzed by dideoxy sequencing (ABI technology) using the ABI3500 at the molecular core facility at the faculty of medicine at AUB as previously described [20]. The results were analyzed at the Congenital Heart Disease Genetics Program (CHDGP) laboratory at the American University of Beirut Medical Center AUBMC.

### Genetic panel selection

**Fig. 1:**
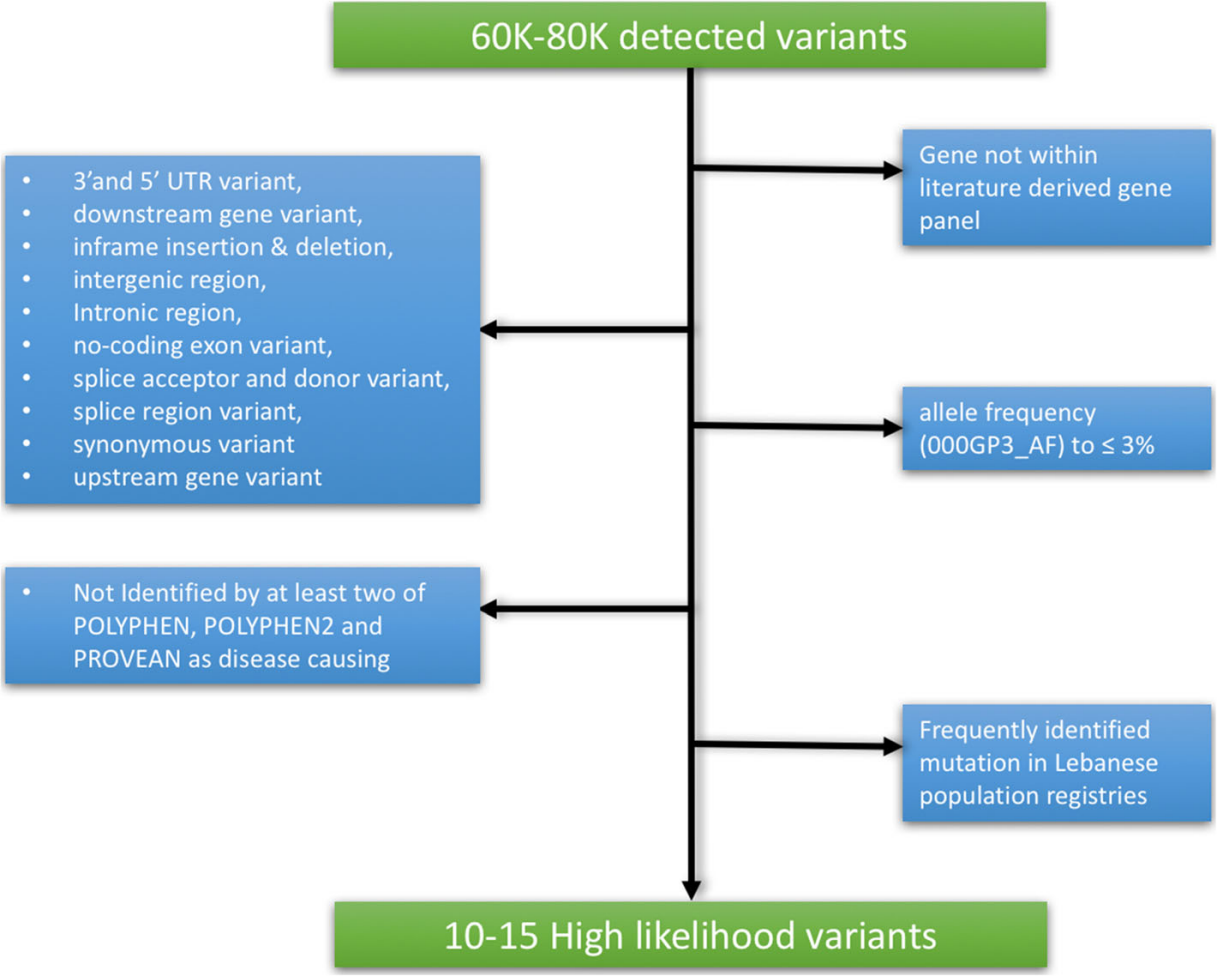
Stepwise strategy for filtering variants to reach 10–15 highly likely disease-causing variants per patient screened. UTR = Untranslated Region; AF = allele frequency

All generated variant call files (vcf) were analyzed according to an extensive virtual gene panel that was chosen to screen for the disease in Lebanon and the region and was adopted from recent published methodology for choosing culprit genes in PAH [21]. Non-synonymous exon coding variants for *BMPR2* were analyzed and a search for compound heterozygous variants associated with the *BMPR2* mutations was performed accordingly using the Illumina variant studio. Non-synonymous missense variants, insertion/deletions variants in the coding regions, and splicing variants with allele frequencies less than 3% were filtered in according to their evolutionary conservation, location within domains and prediction software (SIFT and Polyphen2), while additional variants with no supportive roles were filtered out (Fig. 1).

## Results

### Clinical and demographic profiles of families and patients

A total of 21 patients belonging to 20 different families were recruited in the study from different parts of the country (Table 1). The age of diagnosis as well as additional phenotypes were included, and as previously mentioned, outpatients referred to our center were clinically re-evaluated for confirmation of the phenotype. The cohort includes one family with 2 patients having each hereditary pulmonary arterial hypertension (PAH), 14 patients with idiopathic PAH, and 5 with both PAH and congenital heart disease (CHD). Fifteen out of 21 patients (71%) are females. The idiopathic and hereditary groups had a mean age of 33 years at the time of diagnosis and a mean pulmonary artery pressure of 56 mmHg. Seventeen patients were on dual therapy (sildenafil, and bosentan or masitentan), and 6 patients on sildenafil alone.

**Table 1:** *BMPR2* variants in pulmonary hypertension patients (PHT), with their corresponding global allele frequencies (AF), and predictive protein effects (Polyphen2). ASD (Atrial Septal Defect), VSD (Ventricular Septal Defect), ExAC (Exome Aggregation Consortium), 1000G (1000 Genome from the International Genome Sample Resource)

Family	Sex	Age at Diagnosis	Phenotype	mPAP (mmHg)	Gene	Mutation	familial/ sporadic	Zygosity	ExAC AF	1000 G AF	SIFT	Polyphen2
A	F	5	Large ASD and PAH	45	*BMPR2*	p.Q6*	*s* *(*de novo*)*	Heterozygous			N/A	N/A
B	F	38	PAH	50	*BMPR2*	p.N126S	f	Heterozygous			Damaging	Probably Damaging
C	F	25	PAH	50	*BMPR2*	p.R491W	f	Heterozygous			Damaging	Damaging
	F	25	PAH	94	*BMPR2*	p.R491W	f	Heterozygous			Damaging	Damaging
D	F	11	VSD and PAH	88	*BMPR2*	p.S775 N	s	Heterozygous	0.02516	0.00998403	Tolerated	Benign
E	F	6	ASD and PAH	36	*BMPR2*	p.S775 N	s	Heterozygous	0.02516	0.00998403	Tolerated	Benign
F	M	46	PAH	50	*BMPR2*	p.S775 N	s	Heterozygous	0.02516	0.00998403	Tolerated	Benign
G	M	4	VSD and PAH	43			s					
H	M	11	PAH	77			s					
I	F	1	PAH VSD/ASD	33			s					
J	M	22	PAH	56			s					
K	F	20	PAH	50			s					
L	F	31	PAH	41			s					
M	F	45	PAH	58			s					
N	F	26	PAH	62			s					
O	F	30	PAH	63			s					
P	F	57	PAH	46			s					
Q	F	7	PAH	55			s					
R	F	6	PAH	40			s					
S	M	78	PAH	49			s					
T	M	34	PAH	40			s					

### BMPR2 variants

For all patients, we conducted a primary analysis, whereby only *BMPR2* variants were included. The filtering stringency put a cutoff on all synonymous variants, inframe insertions/deletions, and non-coding ones. Out of the 20 families, we identified six with monoallelic *BMPR2* variants, thus constituting a prevalence of 30%. The phenotypes of the index patients are summarized in Additional file 1: Table S1. Patients with and without *BMPR2* mutations had a similar age of 33 years at diagnosis, and a similar mean pulmonary arterial pressure (PAP) of 57 mmHg. The PAH with CHD group had a mean age of 5.4 years at diagnosis and a mean PAP of 44 mmHg.

**Fig. 2:**
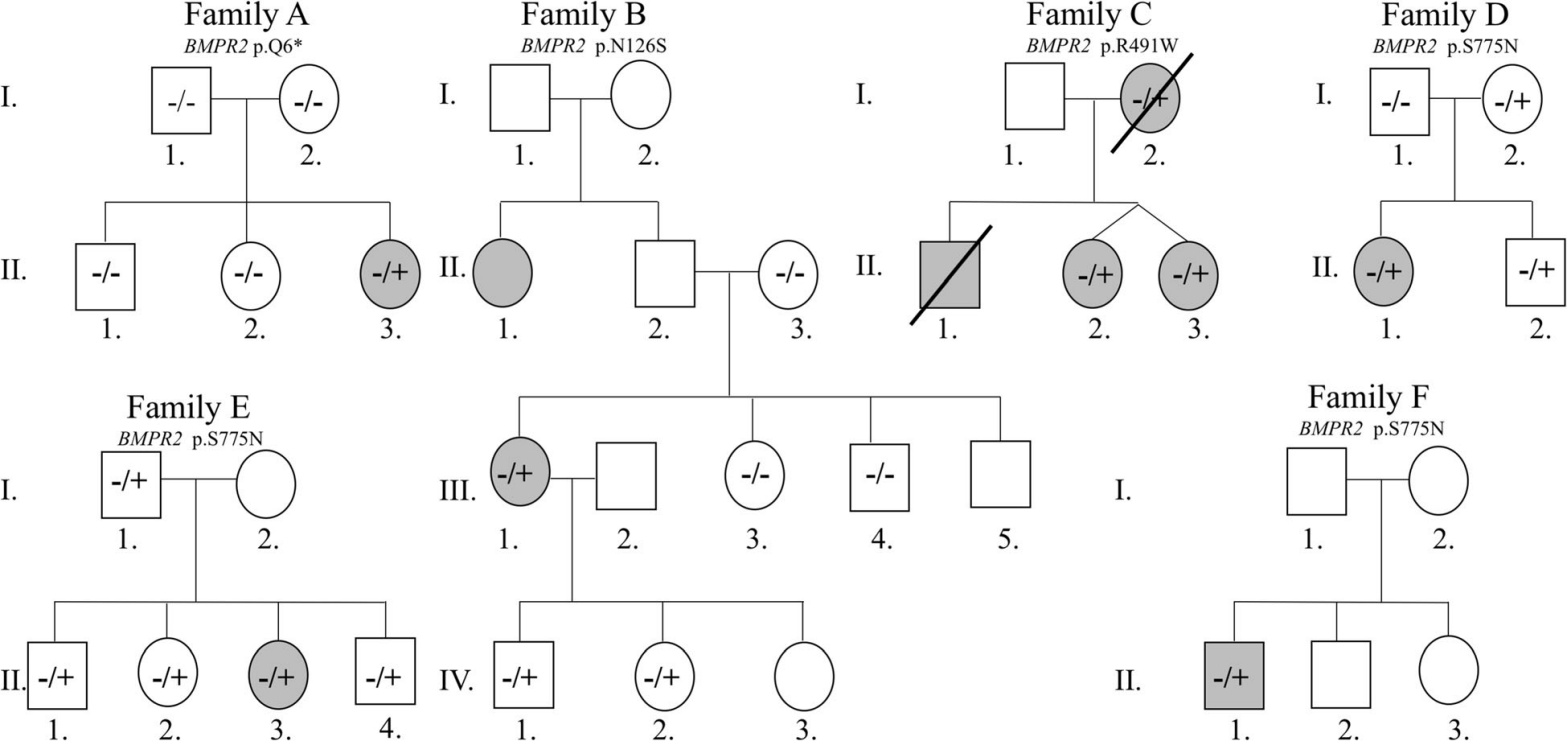
Pedigrees for families showing the different *BMPR2* mutations. The −/− symbol is for normal genes, −/+ is for heterozygous mutations and the +/+ is for homozygous mutations. Square, Male; Circle, female; open symbol, unaffected; filled symbol, affected; symbol with diagonal line, dead individual

Patient AII.3 (Fig. 2), a young girl who had an ASD repair with fenestration at age one, presented at age 5 with dyspnea. She was found to have PAH, with a mean PAP of 45 mmHg. Exome sequencing revealed a heterozygous nonsense mutation within exon 1 of *BMPR2* that leads to a premature stop codon (p.Q6*). Sanger sequencing confirmed the mutation in the affected patient; however, the mutation was not detected in any family members and none of them had pulmonary hypertension on follow-up, suggesting it is a de novo nonsense mutation.

Patient BIII.1 (Fig. 2), a 38-year-old-woman with no apparent relevant family history, presented with progressive dyspnea. She was diagnosed with idiopathic PAH after excluding other possible causes of PAH, with a mean PAP of 50 mmHg. She is being treated with sildenafil and macitentan. Exome sequencing showed a novel missense mutation (p.N126S) within the transmembrane domain of the *BMPR2* receptor. The mutation was reported as probably damaging on the Polyphen-2 and SIFT prediction tools (Table 1). Sanger sequencing confirmed the mutation in the affected individual, but revealed the absence of the mutation in the screened family members. The father (BII.2), who is clinically healthy, refused to participate in the study and may be the source of inheritance (Fig. 2). Interestingly, two of the three children of the indexed patient (namely individuals BIV.1 and BIV.2) also carry the variant (Fig. 2). Although they exhibit no apparent clinical signs or symptoms of PAH, they are still young (< 10 years) compared to their mother (BIII.1) who was diagnosed with PAH at age 38.

Patients CII.2 and CII.3 are 24-year-old female identical twins. Patient CII.2 presented with dyspnea on exertion and syncope. Right heart catheterization (RHC) showed a mean pulmonary artery pressure (PAP) of 94 mmHg and a pulmonary vascular resistance (PVR) of 25 woods units. Acute pulmonary vaso-reactivity testing with inhaled nitric oxide (NO) at a concentration of 40 ppm for 10 min was positive (mean PAP dropped to 21 mmHg). She was NYHA Class III. Her mother had died of idiopathic PAH at age 30 and her younger brother died of PAH at age 8. She was diagnosed with ventricular septal defect (VSD) as a child, which was corrected at age 6. The patient was diagnosed with heritable PAH and she was started on a calcium channel blocker and sildenafil. A repeat RHC, 3 months later, showed a decrease in mean PAP to 54 mmHg and loss of vaso-reactivity response to NO. She improved clinically to NYHA II and bosentan was added to her treatment. Her twin sister CII.3 was asymptomatic and was screened for PAH. She was found to have PAH with a mean PAP of 50 mmHg. There were no signs of VSD. Family C has a previously reported mutation (p.R491W) [22]; it occurs in a highly conserved kinase domain encoded by exon 3 of the gene. Records from the deceased mother and brother showed that the mutation was found in all affected family members CI.2, CII.1, CII.2, and CII.3. The father CI.1 who is phenotypically normal did not participate in the study. The mutation was labeled as “damaging” on Polyphen-2 and SIFT (Table 1, and Fig. 2).

Patients DII.1, EII.3, and FII.1 have the same variant in *BMPR2*, a missense mutation (p.S775 N) previously reported as being disease-causing with very low penetrance [23]. This mutation occurs in the C-terminal domain of the protein within exon 12. It is labeled as benign based on the Polyphen-2 and SIFT prediction tools (Table 1). Patient DII.1 had a small PFO and he developed PAH at age 11. RHC showed a mean PAP of 68 mmHg. He has been stable on sildenafil-bosentan for the last 5 years. Patient EII.3 had an ASD repair at age 1 and developed PAH at age 6 with a mean PAP of 36 mmHg. Patient FII.1 presented at age 46 with dyspnea on exertion. He was diagnosed with idiopathic PAH with no cardiac defects, and his RHC showed a mean PAP of 50.

### Other variants

**Fig. 3:**
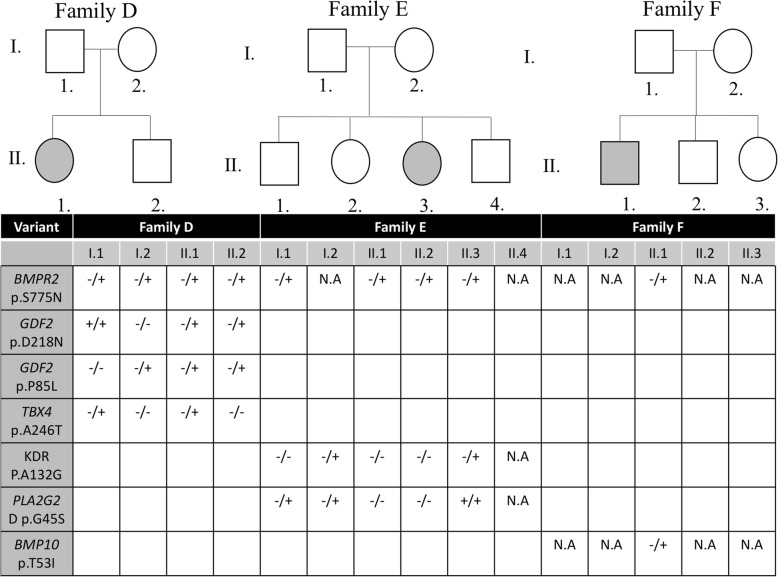
Pedigrees for the p.S775 *BMPR2* mutation in three families with the second hit mutations shown in the table. Square, Male; Circle, female; open symbol, unaffected; filled symbol, affected; symbol with diagonal line, dead individual; the −/− symbol is for normal genes, −/+ is for heterozygous mutations and the +/+ is for homozygous mutations. N/A stands for non-applicable because DNA is not available

In the rest of the families (Additional file 2: Figure S1), we adopted the same stringent filtering mentioned for *BMPR2* on a list of 431 genes curated from the literature and online databases (Table 1). For each patient, a list of variants was extracted (Additional file 1: Table S1). None would explain the phenotype on its own, since there were no homozygous variants in these genes (data not shown). Examples for potential variants are the p.R55W in *BMP4* in family M, and p.M416I variant in *BMP10* in Family L (Additional file 1: Table S1). In contrast, in families with predicted benign and/or low penetrance *BMPR2* mutations (families D, E, and F, Table 1), a search for probable second hit mutations was attempted. The results suggest a potential digenic model for the disease in families D and E (Fig. 3) supported by Sanger sequencing of the involved members in each case. In Family D, biallelic variants in *GDF2* (p.P85L, and p.D218N), and a monoallelic variant in *TBX4* (p.A246T) are novel and are predicted as disease-causing. Those mutations in combination with the “low penetrant” *BMPR2* variant (p.S775 N) could explain the phenotype in the only affected member of the family who is the only one harboring all these variants (Fig. 3, Additional file 1: Table S1). The same applies to Family E, albeit a weak correlation does exist with the p.A1324G variant in *KDR* (Fig. 2, Additional file 1: Table S1).

## Discussion

With our 21 patients, and a worldwide PAH prevalence rate of few dozen per million, our study nearly accounts for all of the recently diagnosed cases in Lebanon, a small country with 4 million inhabitants. The mean age of diagnosis of our cohort of PAH patients is 33 years which is similar to the mean age of diagnosis in other MENA region registries [8, 9], but is significantly lower than the mean age reported in recent European and north American registries [5, 6]. This difference in mean age at diagnosis may be due to differences in survival of PAH patients in our region or due to differences in referral and diagnostic practices. Older patients with pulmonary hypertension have more comorbidities, and are not recognized early enough, if at all,as having PAH. These findings highlight the need for more education in Lebanon and the MENA region to recognize that PAH is no longer a disease affecting only young patients. *BMPR2* mutations are known to be the main genetic causes of familial PAH [24]. To date, more than 300 different mutations have been identified, with 80% of familial cases caused by mutations in this gene [16, 25]. Our results show a predominant *BMPR2* variants rate of 30% that would perfectly align with previous findings, and reinforces the need to look for additional genetic causes, taking into account the implication of environmental factors or compound mutations on the onset of the disease in genetically “pre-disposed” individuals.

Growing evidence implicates the *BMPR2* pathway in structural heart defects. Roberts et al. report *BMPR2* mutations in 6% of patients with diagnosed PAH and congenital defects [26]. Interestingly, three of our probands presented with atrial or ventricular septal defects. A septal defect was also identified in the sister of patient CII.2 with the same mutation (Fig. 2). Mice models of *BMPR2* knock-outs show cardiac anomalies analogous to human septal defects and AV canal congenital heart defects [27–29]. As such, both pulmonary vasculature involvement and hemodynamic stress from increased flow contribute to the pulmonary hypertension in those patients.

### High penetrance mutations: The BMPR2 variants

Deleterious mutations usually lead to a tangible phenotype earlier in the patients’ lives. Patient AII.3 with the *BMPR2* p.Q6* mutation was followed at an early stage after being diagnosed with ventricular septal defect and pulmonary hypertension. His pulmonary artery pressures were higher than would be expected from shunting alone. After surgical correction of the ventricular defect, the pulmonary pressures failed to revert and an underlying cause of the pulmonary hypertension was worked up with likelihood of idiopathic disease. Truncating and/or missense mutations affecting the N-terminal part of the protein have been hypothesized to result in more extensive pulmonary vascular remodeling and more rapid progression of the disease process. This hypothesis is supported by the observation that the worse prognosis associated with a *BMPR2* mutation in patients diagnosed before the age of 30 years is not completely attenuated after adjustment for pulmonary vascular resistance, cardiac index, and vasoreactivity [30]. The early age at presentation reflects the drastic functional role of the mutation. Such a drastic mutation is probably linked to haploinsufficiency with a complete loss of one allele due to mRNA decay rather than a protein with hampered cellular localization and/or function.

Moreover, such deleterious mutations usually occur at functionally important domains within the large 3D structure of the receptor protein. Apart from an early truncation as in patient A.II3, the mutation p.R491W in family C (Fig. 2) occurs at a highly conserved site within the TGF-β superfamily kinase domain of the receptor. Alteration of the Serine/Threonine kinase function hinders the downstream activation cascade within the *BMPR2* pathway. This mutation segregates within the family members defining a high penetrance familial disease.

### Low penetrance and the “second-hit mutation”:

Besides the de novo mutation in *BMPR2*, only two missense monoallelic mutations in *BMPR2* were accounted for in 4 families: p.S775 N and p.N126S. The p.S775 N mutation identified in families D, E, and F has been reported before and is of questionable clinical relevance [23]. Clinical correlation to this mutation has failed to highlight consistent phenotype, as this mutation is common (Minor allele frequency (MAF) on ExAC 0.02516), and when found in patients with pulmonary hypertension it does not associate with age of presentation and vaso-reactivity [23]. As such, the p.S775 N mutation is seen as a possible cause of disease with very low penetrance, perhaps requiring additional genetic or environmental stimuli. The occurrence of the mutation in three patients of this small registry confirms the findings of the literature but raises questions regarding its recurrent significance, especially that in all cases it is inherited from either one of the healthy parents with no ancestry history of PAH. Moreover, the level of pulmonary artery pressures measured in both patients was clinically milder in relation to other *BMPR2* mutations. However, it is important to note here that both patients D and E presented with the disease at a young age implicating a significant commitment of the mutation by environmental or genetic interactions. The affected member in family B harbors a monoallelic inherited missense mutation p.N126S within the extracellular domain of the *BMPR2* protein. Previous studies on mutations within this domain have shown intracellular retention of the receptors and a dampening in SMAD activation [16, 31]. Such functional alteration is critical to the signaling pathway, which might explain the relatively elevated systolic pulmonary artery pressure reading on follow-up echocardiography of the affected individual (data not shown). The fact that in both cases we do not have a clear answer about the pathogenicity of the mutation prompted us to look at other “modifiers” in the pathway leading to PAH involving *BMPR2*. Compound mutations within the *BMPR2* pathway have been hypothesized and recently predicted in the pathogenesis of the disease [32]. This is highlighted more in mutations that are otherwise identified as benign mutations, rendering the analysis of genetic variants trickier. An interesting candidate mutation in patient DII.1 is the *TBX4* variant (p.A246T): this gene has been implicated in small patella syndrome and associated childhood onset pulmonary arterial hypertension [33]. *GDF2, KDR* and *BMP10* mutations modifier moutations are also evident in families D, E and F respectively (Fig. 3). Failure to detect segregating mutations in other families may hint to copy number variants, unknown gene mutations, as well as the possibility of environmental stressors.

One possible link between the genetic basis of penetrance and stressors is wild-type *BMPR2* transcript levels themselves. Hamid et al. demonstrated that the level of production of *BMPR2* transcript and protein by the wild-type allele was associated with disease penetrance [34]. HPAH patients with *BMPR2* mutations had lower transcript levels compared to unaffected mutation carriers [17]. Another possibility is the role of hemodynamic stressors imposed by the associated congenital disease that hasten or activate disease progression in otherwise low penetrance mutations. This is evident in patients D and E. The role of hemodynamic stressors is not very well understood; however, the role of external triggers is well documented in the disease. A trend of earlier presentation of the disease is evident in patients on appetite suppressants [35]. Although such environmental stressors are not central to our cohort, hemodynamic stressors may play a greater role in the failure to sustain sufficient *BMPR*2 transcript levels against a continuous stressor.

## Conclusions

In conclusion, this is the first genetic study by exome sequencing of PAH in the MENA region. The study unveiled novel mutations in *BMPR2*, which may be unique to the MENA region. This shows that more mutations may be found if genetic studies are expanded to other regions and ethnic groups. Our study also hinted at multiple gene interactions being responsible for the development of PAH, which calls for further investigation of the double-hit theory in larger genetic databases. Finally, the study shed light on the limitations of using standardized genetic testing based on North American and European findings in other regions of the world, emphasizing the need for conducting whole exome sequencing (WES) for genetic testing of PAH.

## Additional files

Additional file 1: **Table S1.** Highly suspicious compound mutations per patient linked to PAH molecular pathways. (PDF 670 kb)

Additional file 2: **Figure S1.** Pedigrees for families showing no BMPR2 mutations. Square, Male; Circle, female; open symbol, unaffected; filled symbol, affected; symbol with diagonal line, dead individual. (PDF 752 kb)

## Abbreviations

AUBMC: American University of Beirut Medical Center
*BMPR2*: Bone morphpgenetic protein 2
CHD: Congenital Heart Disease
GDF2: Growth differentiation factor 2
MENA: Middle-East and North Africa
NGS: Next generation sequencing
NYHA: New York Heart Association
PAH: Pulmonary hypertension
PAP: Pulmonary arterial pressure
Vcf: Variant Call File
VSD: Ventricular septal defect
