# Supplementary figures and images for: Clinical and genetic characteristics of pulmonary arterial hypertension in Lebanon
Source: BMC Med Genet. 2018 May 30;19:89. doi: 10.1186/s12881-018-0608-7 (PMC5975525; doi:10.1186/s12881-018-0608-7)

Family G

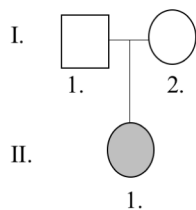

Family H

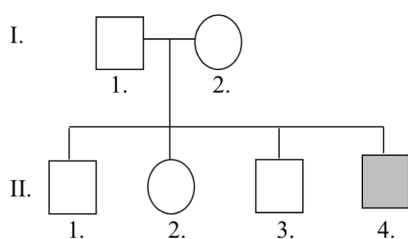

Family I

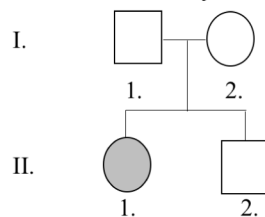

Family J

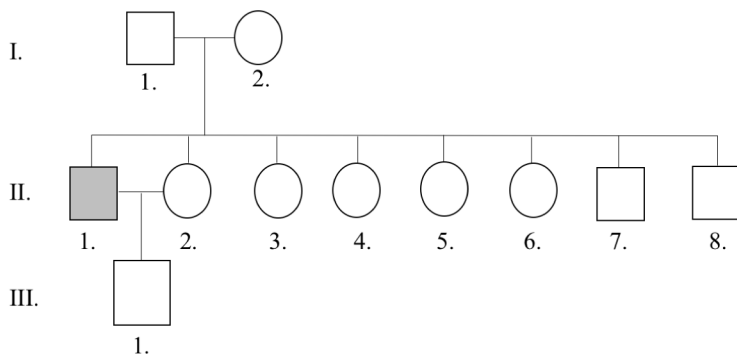

Family K

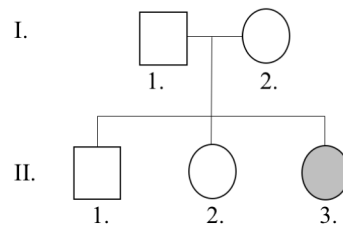

Family L

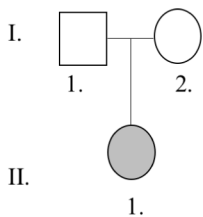

Family M

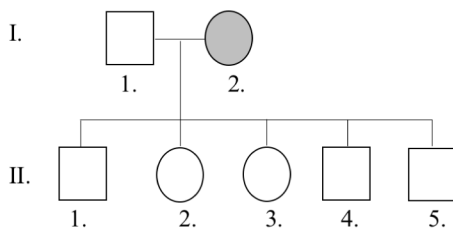

Family P

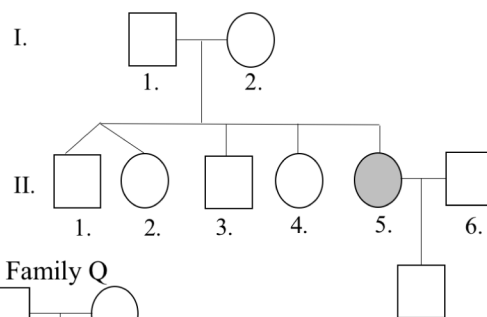

Family O

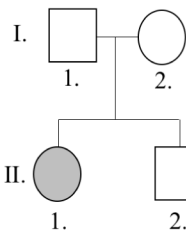

Family N

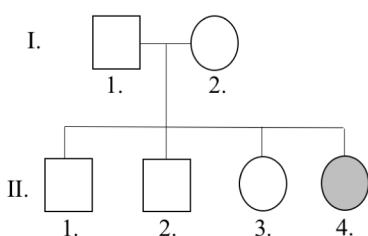

Family Q

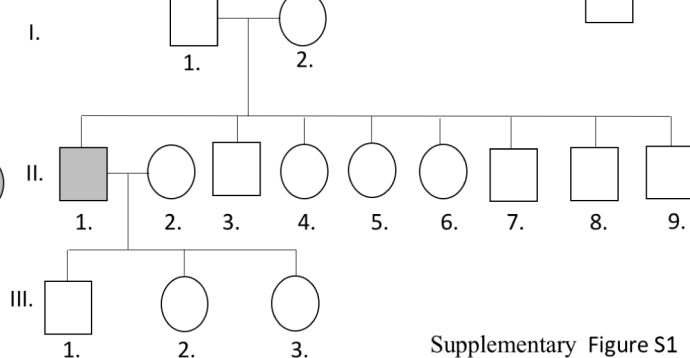

Supplement: Supplementary file 2 — Figure S1. Pedigrees for families showing no BMPR2 mutations. Square, Male; Circle, female; open symbol, unaffected; filled symbol, affected; symbol with diagonal line, dead individual. (PDF 752 kb) [file 12881_2018_608_MOESM2_ESM.pdf]
